# Genomic regions underlying uniformity of yearling weight in Nellore cattle evaluated under different response variables

**DOI:** 10.1186/s12864-018-5003-4

**Published:** 2018-08-16

**Authors:** Laiza Helena de Souza Iung, Herman Arend Mulder, Haroldo Henrique de Rezende Neves, Roberto Carvalheiro

**Affiliations:** 1 School of Agricultural and Veterinarian Sciences, São Paulo State University (Unesp), Via de Acesso Prof. Paulo Donato Castelane, S/N, Vila Industrial, FCAV/UNESP, Jaboticabal, São Paulo, 14884-900 Brazil; 2 Wageningen University & Research Animal Breeding and Genomics, P.O. Box 338, 6700 AH Wageningen, The Netherlands; 3 GenSys Consultores Associados S/S Ltda, Porto Alegre, RS 90680-000 Brazil

**Keywords:** Beef cattle, DHGLM, Genetic heterogeneity of residual variance, Growth traits, GWAS, Micro-environmental sensitivity

## Abstract

**Background:**

In livestock, residual variance has been studied because of the interest to improve uniformity of production. Several studies have provided evidence that residual variance is partially under genetic control; however, few investigations have elucidated genes that control it. The aim of this study was to identify genomic regions associated with within-family residual variance of yearling weight (YW; *N* = 423) in Nellore bulls with high density SNP data, using different response variables. For this, solutions from double hierarchical generalized linear models (DHGLM) were used to provide the response variables, as follows: a DGHLM assuming non-null genetic correlation between mean and residual variance (r_mv_ ≠ 0) to obtain deregressed EBV for mean (dEBV_m_) and residual variance (dEBV_v_); and a DHGLM assuming r_mv_ = 0 to obtain two alternative response variables for residual variance, dEBV_v_r0_ and log-transformed variance of estimated residuals (ln_${\upsigma}_{\widehat{\mathrm{e}}}^2$).

**Results:**

The dEBV_m_ and dEBV_v_ were highly correlated, resulting in common regions associated with mean and residual variance of YW. However, higher effects on variance than the mean showed that these regions had effects on the variance beyond scale effects. More independent association results between mean and residual variance were obtained when null r_mv_ was assumed. While 13 and 4 single nucleotide polymorphisms (SNPs) showed a strong association (Bayes Factor > 20) with dEBV_v_ and ln_${\upsigma}_{\widehat{\mathrm{e}}}^2$, respectively, only suggestive signals were found for dEBV_v_r0_. All overlapping 1-Mb windows among top 20 between dEBV_m_ and dEBV_v_ were previously associated with growth traits. The potential candidate genes for uniformity are involved in metabolism, stress, inflammatory and immune responses, mineralization, neuronal activity and bone formation.

**Conclusions:**

It is necessary to use a strategy like assuming null r_mv_ to obtain genomic regions associated with uniformity that are not associated with the mean. Genes involved not only in metabolism, but also stress, inflammatory and immune responses, mineralization, neuronal activity and bone formation were the most promising biological candidates for uniformity of YW. Although no clear evidence of using a specific response variable was found, we recommend consider different response variables to study uniformity to increase evidence on candidate regions and biological mechanisms behind it.

**Electronic supplementary material:**

The online version of this article (10.1186/s12864-018-5003-4) contains supplementary material, which is available to authorized users.

## Background

Uniformity is becoming increasingly important among livestock species. In meat production systems, this is more evident by the increasing adoption of economic incentives by slaughterhouses to stimulate farmers to deliver animals that meet specific carcass standards. If uniformity is partly under genetic control, genetic selection could be used to improve uniformity of animals. Genetic control of uniformity can be the result of how genotypes respond differently towards unknown micro-environmental factors [1, 2]. Such a phenomenon is called genetic heterogeneity of residual variance or genetic variance in micro-environmental sensitivity. Up to now, several studies support the existence of a genetic component on residual variance and draw attention for its potential to improve uniformity through selection (e.g. [3–6]). Unraveling the genetic basis of heterogeneity of residual variance through genome-wide association studies (GWAS) will help to understand the biology behind it and increase selection response by identifying candidate genes affecting uniformity and including them in genomic prediction.

Few GWAS for uniformity traits have been performed in livestock and there is no consensus about the most appropriate phenotype to be used in such studies. Phenotypic standard deviation and coefficient of variation was used to address uniformity of egg weight in chickens by Wolc et al. [7] and birth weight in pigs by Wang et al. [8, 9]. Residual variance per individual from double hierarchical generalized linear model (DHGLM; obtained according to Rönnegård et al. [10]) were used as response variable by Mulder et al. [11] to identify genomic regions related to residual variance of somatic cell score in dairy cattle. Estimated breeding values (EBV) from a DHGLM, using an extension developed by Felleki et al. [12], were deregressed (dEBV) and used to identify genomic regions associated with variability of litter size in pigs by Sell-Kubiak et al. [13]. Such variety of phenotypes used as response variables can be explained because uniformity can be measured in different ways depending on trait and data structure. Statistical analysis requires either the within-individual variance based on repeated observation per animal or the within-family variance based on large offspring groups per family. Furthermore, the availability of genotypes influences the choice for the response variable as well. The choice for dEBV in the study by Sell-Kubiak et al. [13] was made because of availability of genotypes on boars with many phenotyped offspring and sows with genotypes and phenotypes for litter size. In the case of Mulder et al. [11], genotypes were only available on cows with many phenotypic observations on experimental farms.

In beef cattle, growth traits are often used as selection criteria in breeding programs due to its economic impact in meat production. Several genes and genomic regions influencing the mean of these traits have been identified (e.g. [14–16]). In addition, the existence of a genetic component on residual variance of growth traits was previously observed [17–19]. However, no further investigations have been carried out to study the genetic mechanisms underlying uniformity of growth traits. Thus, the aim of this study was to identify genomic regions associated with within-family residual variance of yearling weight (YW) in Nellore cattle through GWAS, using different response variables, and candidate genes to better understand the biology behind genetic control of uniformity.

## Results

Our aim was to identify genomic regions (1-Mb windows among the top 20 that explained the largest proportion of genetic variance shared between response variables and 1-Mb windows with SNPs that showed a strong association by BF) associated with within-family residual variance of YW in Nellore cattle, using different response variables in GWAS. For this, we used solutions from DHGLM assuming: i) non-null genetic correlation between mean and residual variance of YW (r_mv_ ≠ 0) to obtain deregressed EBV for mean (dEBV_m_) and residual variance (dEBV_v_); and ii) r_mv_ = 0 to obtain dEBV_v_r0_ and log-transformed variance of estimated residuals (ln_${\upsigma}_{\widehat{\mathrm{e}}}^2$).

The Pearson’s correlations between the different response variables and the number of common 1-Mb windows (among the top 20 that explained the largest proportion of genetic variance in the association studies) among them are shown in Table 1. As expected, dEBV_m_ was highly correlated with dEBV_v_, 0.90, given the high and positive r_mv_ (0.76; [19]). As a result, eight out of the top 20 windows were shared between dEBV_m_ and dEBV_v_: chromosome (Chr) 1 (92 Mb; Chr1_92), 3 (Chr3_42 and Chr3_45), 13 (Chr13_59), 14 (Chr14_24 to 26) and 16 (Chr16_21) (Table 2). The dEBV_m_ was also positively correlated with dEBV_v_r0_, 0.19, but no overlap was found among the top 20 windows.

**Table 1:** Number of common 1-Mb windows^a^ (above diagonal) and Pearson’s correlation (below diagonal) between response variables

	dEBV_m_	dEBV_v_	dEBV_v_r0_	ln_${\upsigma}_{\widehat{\mathrm{e}}}^2$
dEBV_m_		8	0	0
dEBV_v_	0.90 (0.02)		1	1
dEBV_v_r0_	0.19 (0.05)	0.53 (0.04)		2
ln_${\upsigma}_{\widehat{\mathrm{e}}}^2$	−0.01 (0.05)	0.08 (0.05)	0.24 (0.05)	

^a^ Considering only the top 20 windows that explained the largest proportion of genetic variance for each response variable; dEBV_m_ and dEBV_v_: deregressed EBV for mean and residual variance of yearling weight, respectively; dEBV_v_r0_ and ln_${\upsigma}_{\widehat{\mathrm{e}}}^2$: deregressed EBV for residual variance and log-transformed variance of estimated residuals, respectively, both assuming null genetic correlation between mean and residual variance. Standard errors are presented between brackets

**Table 2:** Common 1-Mb windows shared by deregressed EBV for mean (dEBV_m_) and residual variance (dEBV_v_)

Windows^a^	% Var^b^		Candidate gene and/or QTL region^c^	Reference^d^
	dEBV_m_	dEBV_v_		
Chr1_92	2.57	0.51	Chr1_70.2:94.6, Chr1_87.2:98.9	[20, 21]
Chr3_42	2.89	1.54	Chr3_41	[15]
Chr3_45	1.00	1.65	Chr3_41	[15]
Chr13_59	1.07	0.60	Chr13_58, *GNAS*	[15, 101]
Chr14_24:26	3.76	3.07	RB1CC1, NPBWR1, PLAG1, PENK	[14–16, 23–27]
Chr16_21	1.26	5.13	Chr16_8.4:34, Chr16_10.6:22.5, Chr16_19.4:40	[21, 22]

In bold are the windows harboring strong associations (Bayes Factor > 20) for both response variables

^a^ Represented by chromosome (Chr) and physical position (in Mb) (Chr_Mb and Chr_Mb:Mb)

^b^ Proportion of genetic variance explained by each 1-Mb window

^c^ Ensembl Database UMD3.1 and QTLdb database (represented by Chr_Mb and Chr_Mb:Mb)

^d^ Previous studies reporting QTL regions and/or candidate genes related with growth traits that are next or within the windows found in this study

The response variables for residual variance of YW had low to moderate correlations with each other (Table 1). The largest correlation was observed between dEBV_v_ and dEBV_v_r0_ (0.53), which shared one window among the top 20, Chr12_5. On the other hand, the smallest correlation was between dEBV_v_ and ln_${\upsigma}_{\widehat{\mathrm{e}}}^2$ (0.08), which also shared only one window among the top 20, Chr22_52. The dEBV_v_r0_ and ln_${\upsigma}_{\widehat{\mathrm{e}}}^2$ showed a correlation of 0.24 and had two common regions among the top 20: Chr6_105 and Chr9_8.

**Fig. 1:**
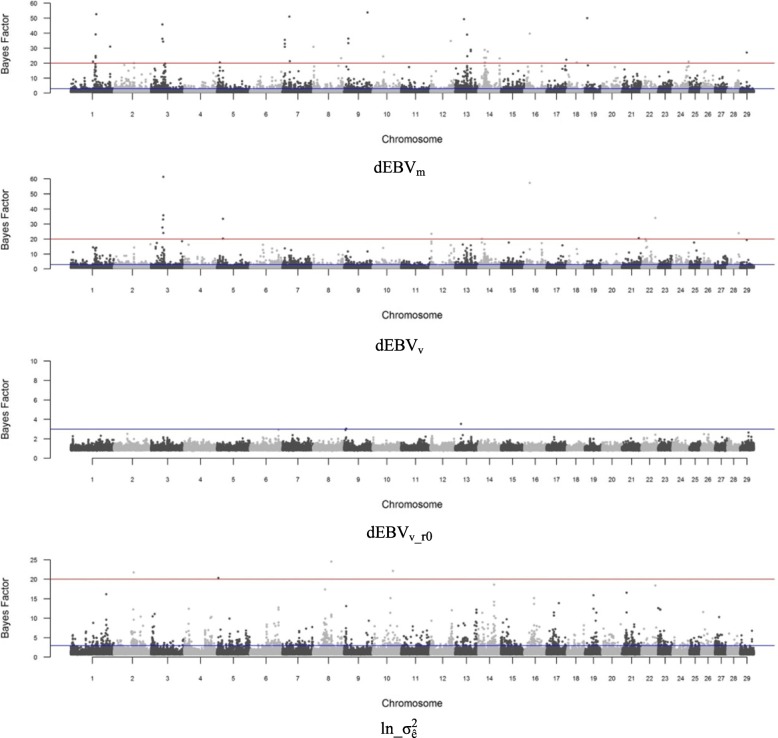
Manhattan plot for the mean (dEBV_m_) and residual variance (dEBV_v_, dEBV_v_r0,_ ln_${\upsigma}_{\widehat{\mathrm{e}}}^2$) of yearling weight. dEBV_m_ and dEBV_v_: deregressed EBV for mean and residual variance, respectively; dEBV_v_r0_ and ln_${\upsigma}_{\widehat{\mathrm{e}}}^2$: deregressed EBV for residual variance and log-transformed variance of estimated residuals, respectively, both obtained from solutions of a DHGLM assuming null genetic correlation between mean and residual variance. The horizontal blue and red lines represent Bayes Factor of 3 (suggestive) and 20 (strong), respectively

**Fig. 2:**
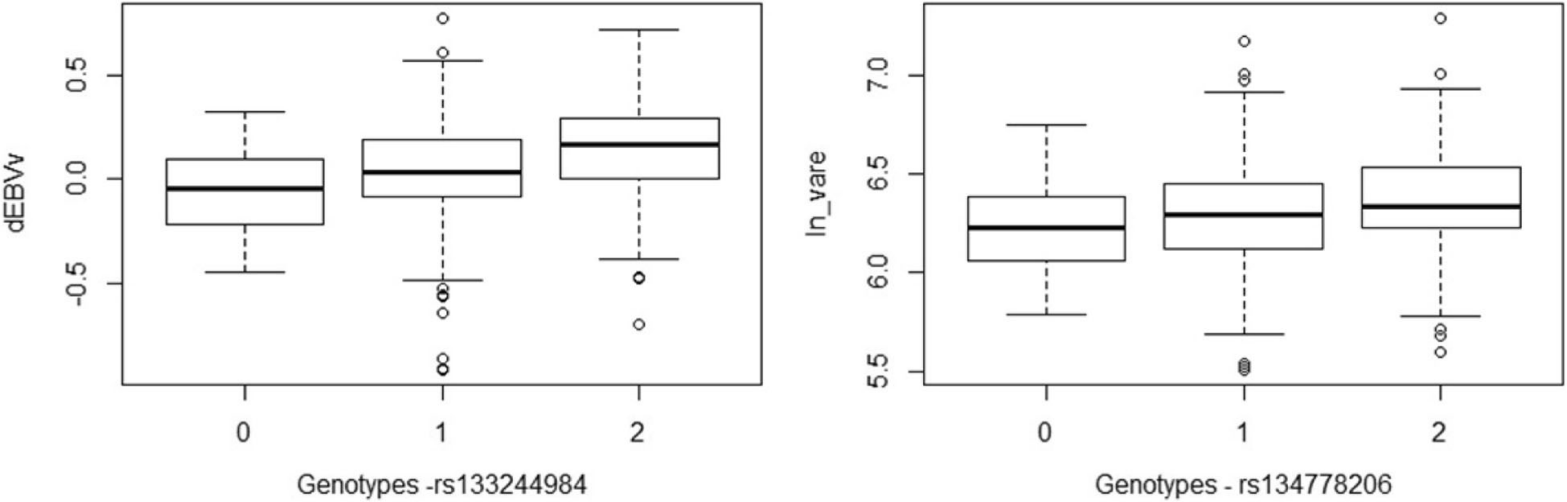
Box plots for the dEBV_v_ and ln_${\upsigma}_{\widehat{\mathrm{e}}}^2$ according to rs133244984 and rs134778206 genotypes, respectively

Based on the GWAS results, 40, 13 and 4 single nucleotide polymorphisms (SNPs) showed a strong association (BF > 20) with dEBV_m_, dEBV_v_ and ln_${\upsigma}_{\widehat{\mathrm{e}}}^2$, respectively (Fig. 1 and Additional file 1). Box plots of dEBV_v_ and ln_${\upsigma}_{\widehat{\mathrm{e}}}^2$ by genotype of the markers with higher BF were presented to give an overview about the signals that these SNPs are capturing (Fig. 2). In both cases, the first homozygous genotype (0) was more uniform, with lower mean and lower dispersion of the corresponding response variable, compared to the other genotypes, although AA was less frequent in relation to AB and BB. Only a few suggestive association signals (2; BF > 3) were found for dEBV_v_r0_. All these SNPs are located within the top 20 1-Mb windows that explained the largest proportion of genetic variance in each response variable. The top 20 windows explained together 34.6, 32.6, 4.4 and 20.0% of the genetic variance of dEBV_m_, dEBV_v,_ dEBV_v_r0_ and ln_${\upsigma}_{\widehat{\mathrm{e}}}^2$ (data not shown). The remaining part of genetic variance was explained by the remaining windows, those not included in the top 20. The range of the proportion of variance explained by individual windows and their sum suggest that uniformity of YW behaves as a polygenic trait determined by several genes, as well as its mean.

Within the common windows shared by dEBV_m_ and dEBV_v_, genes and QTL regions were mapped (Table 2). Several candidate genes for growth traits in beef cattle were identified on chromosome 14 (*RB1CC1, NPBWR1, PLAG1* and *PENK*). Such findings highlight that most of the effects captured by dEBV_v_ can be due to the strong r_mv_ and could be potentially scale effects [19].

In order to prove that these common regions can really affect the variance beyond a simple scale effect, we applied a similar procedure as described in Wolc et al. [7]. For this, only the common SNP with the highest BF in each window was considered. The absolute effect of these SNPs on dEBV_m_ and dEBV_v_ were divided by the mean of YW and mean of ${\upsigma}_{\mathrm{e}}^2$, respectively. If SNP would have an effect on mean and variance due to scaling, then the standardized effects should be the same or higher on mean. However, all SNPs showed a greater standardized effect on dEBV_v_ than dEBV_m_ indicating that these regions affect the residual variance beyond a simple scale effect. These results suggest that even with a high and strong r_mv_ it is possible to find regions determining mean growth and its variability simultaneously, while the effects on the variance are larger than can be explained by a simple scale effect.

In the shared windows by dEBV_v_r0_ and ln_${\upsigma}_{\widehat{\mathrm{e}}}^2$, Chr6_105 and Chr9_8, genes involved in metabolism (*NUDT9*, *SPARCL1* and *MAN2B2*), mineralization (*DMP1* and *DSPP*) and neuronal activity (*PPP2R2C, JAKMIP1* and *ADGRB3*) were considered potential candidates for uniformity (Table 3). No gene was identified within Chr12_5, shared by dEBV_v_ and dEBV_v_r0_. However, several promising genes were found on Chr22_52 shared by dEBV_v_ and ln_${\upsigma}_{\widehat{\mathrm{e}}}^2$; among them, genes related to metabolism (e.g. *DPYD*, *AK7, GMPPB, AMT* and *GLUD1),* stress *(IP6K2* and *UCN2)* and inflammatory and immune responses *(UBA7, RNF123, MST1* and *RHOA)* (Table 3)*.*

**Table 3:** Overlapped 1-Mb windows between response variables for uniformity (dEBV_v_, dEBV_v_r0_ and ln_${\upsigma}_{\widehat{\mathrm{e}}}^2$)

Windows^a^	Shared by			% Variance Explained^b^ (BF^c^)			Candidate Genes^d^
	dEBV_v_	dEBV_v_r0_	ln_${\upsigma}_{\widehat{\mathrm{e}}}^2$	dEBV_v_	dEBV_v_r0_	ln_${\upsigma}_{\widehat{\mathrm{e}}}^2$	
Chr12_5	X	X		1.69 (23.48)	0.19 (1.99)		–
Chr22_52	X		X	1.73 (33.97)		0.72 (18.37)	UBA7, IP6K1, GMPPB, RNF123, MST1, APEH, AMT, RHOA, USP4, USP19, IMPDH2, NDUFAF3, SLC25A20, PRKAR2A, IP6K2, UQCRC1, UCN2, PFKFB4, SHISA5
Chr6_105		X	X		0.29 (2.94)	0.91 (12.74)	NUDT9, SPARCL1, DSPP, DMP1, MAN2B2, PPP2R2C, JAKMIP1
Chr9_8		X	X		0.28 (3.05)	0.67 (13.08)	ADGRB3

dEBV_v_: deregressed EBV for residual variance of yearling weight; dEBV_v_r0_ and ln_${\upsigma}_{\widehat{\mathrm{e}}}^2$: deregressed EBV for residual variance and log-transformed variance of estimated residuals, respectively, both assuming null genetic correlation between mean and residual variance

^a^ Represented by chromosome (Chr) and physical position (in Mb) (i.e. Chr_Mb)

^b^ Proportion of genetic variance in each 1-Mb window

^c^ Single nucleotide polymorphism (SNP) with the highest Bayes Factor (BF) within each 1-Mb window

^d^ Ensembl Database UMD3.1. See the complete gene list in each window in Additional file 1

For uniformity, promising genes related to metabolism: *ATP6V1B2* (Chr8), *LPL* (Chr8), *OSBPL8* (Chr5) and *GLI2* (Chr2); immune response: *GLIPR1* (Chr5), neuronal plasticity: *LZTS1* (Chr8) and stress response: *SLC18A1* (Chr8), *HIF1A* (Chr10)*, BBS10* (Chr5) and *CLASP1* (Chr2) were observed in the windows of the SNPs that showed strong association with ln_${\upsigma}_{\widehat{\mathrm{e}}}^2$ (Table 4). For dEBV_v_, most of the genes in the regions harboring SNPs that showed strong association were related to metabolism: *DPYD* (Chr3), *BTG1* (Chr5), *AK7* (Chr21), *ADIRF* (Chr28)*, GLUD1* (Chr28), *IP6K1, GMPPB, APEH, AMT, USP4, USP19, IMPDH2, NDUFAF3, SLC25A20, PRKAR2A, UQCRC1, PFKFB4* and *SHISA5* all located on Chr22 (Table 5). In addition, genes involved in stress response: *BDKRB2* and *BDKRB1* (Chr21), and *IP6K2* and *UCN2* (Chr22); inflammatory and immune responses: *EEA1* (Chr5), *TCL1A* and *TCL1B* (Chr21), *UBA7*, *RNF123*, *MST1* and *RHOA* all on Chr11*;* neuronal plasticity: *PLPPR4* and *PLPPR5* (Chr3); and bone formation: *BMPR1A* (Chr28) were also found (Table 5). In summary, most of the regions associated with residual variance of YW harbor interesting candidate genes related to metabolism, stress, inflammatory and immune responses, mineralization, neuronal activity and bone formation.

**Table 4:** Significant SNPs^a^ associated with log-variance of estimated residuals (ln_${\upsigma}_{\widehat{\mathrm{e}}}^2\Big)$

SNP	Chr	Position (Mb)	BF	MAF	SNP effect	Windows^b^	% Var^c^	Candidate Genes^d^
rs134778206	8	67.46	24.51	0.40	0.00075	Chr8_68	2.34	LPL, SLC18A1, ATP6V1B2, LZTS1
rs133984762	10	74.34	22.11	0.48	0.00068	Chr10_75	1.27	HIF1A
rs132792897	2	73.42	21.75	0.50	−0.00066	Chr2_74	1.30	GLI2, CLASP1
rs135481650	5	5.38	20.32	0.49	−0.00061	Chr5_6	1.04	BBS10, OSBPL8

^a^ Single nucleotide polymorphisms (SNPs) that showed a strong association with ln_${\upsigma}_{\widehat{\mathrm{e}}}^2$ according Bayes Factor (BF > 20)

^b^ Represented by chromosome (Chr) and physical position (in Mb) (i.e. Chr_Mb)

^c^ Proportion of genetic variance in each 1-Mb window

^d^ Ensembl Database UMD3.1. See the complete gene list in each window in Additional file 1

**Table 5:** Significant SNPs^a^ associated with deregressed EBV for residual variance (dEBV_v_)

SNP	Chr	Position (Mb)	BF	MAF	SNP effect	Windows^b^	% Var^c^	Candidate Genes^d^
rs133244984	3	45.30	61.24	0.32	0.00221	Chr3_46	8.67	DPYD
rs137051934	16	20.84	57.29	0.40	0.00190	Chr16_21	5.13	–
rs135408640	3	45.29	35.74	0.41	0.00117	Chr3_46	8.67	DPYD
rs42014753	22	51.60	33.97	0.37	0.00112	Chr22_52	1.73	UBA7, IP6K1, GMPPB, RNF123, MST1, APEH, AMT, RHOA, USP4, USP19, IMPDH2, NDUFAF3, SLC25A20, PRKAR2A, IP6K2, UQCRC1, UCN2, PFKFB4, SHISA5
rs110480161	5	22.01	33.44	0.45	0.00110	Chr5_23	2.38	BTG1
rs136349671	3	44.37	32.98	0.39	0.00106	Chr3_45	1.65	PLPPR4, PLPPR5
rs137143404	3	41.54	27.60	0.50	−0.00087	Chr3_42	1.54	–
rs135488380	3	45.34	23.95	0.34	0.00079	Chr3_46	8.67	DPYD
rs137135953	28	41.34	23.88	0.37	0.00074	Chr28_42	0.84	BMPR1A, ADIRF, GLUD1
rs133451489	12	4.46	23.48	0.39	−0.00072	Chr12_5	1.69	–
rs109757968	21	62.41	20.46	0.33	0.00065	Chr21_63	0.67	TCL1B, TCL1A, BDKRB2, BDKRB1, AK7
rs136908466	5	22.06	20.28	0.43	−0.00063	Chr5_23	2.38	BTG1
rs137014141	14	14.47	20.16	0.35	0.00062	Chr14_15	0.85	–

^a^ Single nucleotide polymorphisms (SNPs) that showed a strong association with ln_${\upsigma}_{\widehat{\mathrm{e}}}^2$ according Bayes Factor (BF > 20)

^b^ Represented by chromosome (Chr) and physical position (in Mb) (i.e. Chr_Mb)

^c^ Proportion of genetic variance in each 1-Mb window

^d^ Ensembl Database UMD3.1. See the complete gene list in each window in Additional file 1

## Discussion

In this study, we identified genomic regions associated with within-family residual variance of YW in Nellore cattle by using different response variables (dEBV_v_, dEBV_v_r0_ and ln_${\upsigma}_{\widehat{\mathrm{e}}}^2$). Thirteen and four strong associations (BF > 20) were observed using dEBV_v_ and ln_${\upsigma}_{\widehat{\mathrm{e}}}^2$, respectively. Overlapping windows among the top 20 that explained most of the genetic variance were observed between dEBV_v_ and the other response variables including dEBV_m_. The dEBV_v_ shared 8 out of the top 20 windows with dEBV_m_, one with dEBV_v_r0_ and another one with ln_${\upsigma}_{\widehat{\mathrm{e}}}^2$. Potential candidate genes related to metabolism (carbohydrate, energy, lipid, nucleotide and amino acid), stress, inflammatory and immune responses, mineralization, neuronal activity and bone formation were found for ln_${\upsigma}_{\widehat{\mathrm{e}}}^2$ and dEBV_v_.

### Common regions between dEBV_m_ and dEBV_v_

All common regions shared by dEBV_m_ and dEBV_v_ contained previously found QTL, associated with birth, yearling and carcass weight in other breeds [15, 20–22]. The region found on chromosome 14 (24 to 26 Mb) was associated with growth, feed efficiency and carcass quality traits in beef cattle and several candidate genes were reported [14–16, 23–27]. According to these studies, pleiomorphic adenoma gene 1 (*PLAG1*) seems to be the major gene due to its role in regulating insulin-like growth factors (IGF; [28]). However, some of these genes, like RB1-inducible coiled-coil 1 (*RB1CC1*), neuropeptides B/W receptor 1 (*NPBWR1*) and proenkephalin (*PENK*), are also involved in processes that can contribute to determine uniformity of growth traits, like YW. *RB1CC1* is involved in cell growth and differentiation, senescence, apoptosis and autophagy, and was one of the genes differentially expressed in heat stressed chickens [29, 30]. *NPBWR1* modulates feeding behavior and energy homeostasis and *PENK* is responsible for producing enkephalins in response to stress [31, 32]. Based on these findings and the fact that these shared regions between dEBV_m_ and dEBV_v_ showed effects beyond scale effects, we can conclude that the same gene can affect both, mean and its variability.

### Promising genes associated with ln_${\upsigma}_{\widehat{\mathrm{e}}}^2$, dEBV_v_ and dEBV_v_r0_

Genes involved in metabolism (*ATP6V1B2, LPL, OSBPL8* and *GLI2*), immune response (*GLIPR1*), neuronal plasticity (*LZTS1*) and stress response (*SLC18A1, HIF1A* and *BBS10* and *CLASP1*) were considered the most promising biological candidates for uniformity using ln_${\upsigma}_{\widehat{\mathrm{e}}}^2$. For dEBV_v_, genes related to metabolism (*DPYD, BTG1, AK7*, *ADIRF, GLUD1*, *IP6K1, GMPPB, APEH, AMT, USP4, USP19, IMPDH2, NDUFAF3, SLC25A20, PRKAR2A, UQCRC1, PFKFB4* and *SHISA5*), stress response (*BDKRB2, BDKRB1, IP6K2* and *UCN2),* inflammatory and immune responses (*TCL1A, TCL1B*, *UBA7*, *RNF123*, *MST1* and *RHOA),* neuronal plasticity (*PLPPR4* and *PLPPR5)* and bone formation (*BMPR1A*) were identified as potential candidates. Such findings support partially the hypothesis that most likely the mean of a trait and its uniformity share processes, like those related to metabolism. However, uniformity may be a trait which is even more complex and may be controlled by several processes and mechanisms beyond metabolism, in order to reach homeostasis and maintain the body in balance.

Among the metabolism-related genes for ln_${\upsigma}_{\widehat{\mathrm{e}}}^2$*,* ATPase, H+ transporting, V1 subunit B2 (*ATP6V1B2*) encodes a component of the vacuolar ATPase (V-ATPase), which is activated according to the energy level, like during glucose starvation [33]. Previously, the *ATP6V1B2* gene was found to be associated with feed efficiency traits in beef cattle [34]. Lipoprotein lipase (*LPL*) and oxysterol binding protein-like 8 (*OSBPL8*) genes are involved in lipid metabolism. *LPL* is responsible for hydrolysis of circulating triglycerides and very low-density lipoprotein [35]. Associations between *LPL* and growth and carcass quality traits were also reported previously [36–39]. *OSBPL8* modulates lipid homeostasis through sterol regulatory element binding proteins (SREBPs; [40]). In addition, glioma-associated oncogene family zinc finger 2 (*GLI2*) gene is a transcription factor of the Hedgehog (Hh) signaling pathway, which is involved in muscle development mainly during embryonic development, but also in adult tissue homeostasis and repair [41, 42].

For dEBV_v_, most of the genes are involved in metabolic pathways or related processes to it, such as metabolism of carbohydrate, energy and lipid, nucleotide and amino acid (*GMPPB, UQCRC1, PFKFB4, AMT, GLUD1*, *AK7, IMPDH2, NDUFAF3, DPYD, SLC25A20, PRKAR2A* and *IP6K1)*. Genes regulating muscle cell proliferation, differentiation and myogenesis (*BTG1* [43], *USP4* and *USP19* [44, 45], and *SHISA5*), adipogenic differentiation (*ADIRF* [46]) and proteolysis (*APEH*) also showed metabolic functions that potentially can contribute to uniformity of YW.

Genes involved in the energy and protein metabolism may play an important role to keep body homeostasis against environmental perturbations. Usually, metabolic responses to these stressors involve catabolic processes such as energy mobilization and protein degradation [47, 48]. In such situations, genes related to metabolism are required to recover homeostasis and consequently part of the energy is diverted from growth which can potentially affect performance and therefore explain the potential trade-off between energy for growth and energy for homeostasis.

Genes related to stress response were also found using ln_${\upsigma}_{\widehat{\mathrm{e}}}^2$ *(SLC18A1, HIF1A, BBS10* and *CLASP1)* and dEBV_v_ (*BDKRB2, BDKRB1, IP6K2* and *UCN2*). Stress is a response to new environmental conditions, like nutrition, housing or any stimuli, that threatens homeostasis. Therefore, stress genes may have a direct effect on uniformity, such that animals that are worse in dealing with environmental perturbations tend to be less uniform (e.g. [49]).

Vesicular monoamine transporter 1 (*SLC18A1*) and urocortin 2 (*UCN2*) genes are involved in the hypothalamic-pituitary-adrenal (HPA) axis. *SLC18A1* acts in the final stage of the HPA-axis, transporting catecholamines like dopamine, noradrenaline and adrenaline. Catecholamines are released as a physiological stress response [50]. *UCN2* is a member of the corticotropin-releasing hormone (CRH) family, which is a key mediator of the stress response by activating the HPA axis [51, 52].

The hypoxia-inducible factor 1-alpha *(HIF1A*) gene encodes a transcription factor that is induced by hypoxia, promoting angiogenesis, cell proliferation/survival and glucose/iron metabolism [53]. Angiogenesis is the process of new vessel formation, which is related to heat stress. Recently, *HIF1A* was highly expressed in the ruminal epithelium of highly-efficient animals [54], which can be explained by its functions, mainly angiogenesis and glucose metabolism. It indicates that animals can differ in relation to absorption and energy generation, changing individual growth performance.

The Bardet-Biedl Syndrome 10 (*BBS10*) gene, a member of heat shock protein (HSP) family, encodes a chaperonin-like protein [55]. The HSP family plays a crucial role in response to several stressful conditions in addition to heat stress [56], and it is not the first time that a member of this family is related to uniformity. Previously, *Hsp90* was considered a candidate gene for developmental stability of morphological traits in *Drosophila melanogaster* and *Arabidopsis thaliana* (e.g. [57–60]) and variability of litter size in pigs [13]. It supports the evidence that the function of chaperones is extended to several organisms, making these HSP protein genes promising candidates for uniformity.

Another gene related to HSP family is the inositol hexakisphosphate kinase 2 (*IP6K2*), a metabolism related-gene that also plays a role in apoptosis [61]. However, its catalytic activity is regulated by *HSP90* gene [62], which suggests that cellular response can also be expected due to environmental stimuli.

Cytoplasmic linker associated protein 1 (*CLASP1*) and bradykinin receptor B1 and B2 (*BDKRB1* and *BDKRB2)* genes are involved in regulation of wound healing and blood pressure regulation, mechanisms that can be used to deal with environmental stressors. Previously, Coble et al. [63] found *BDKRB1* among up-regulated genes during heat stress in chickens.

Some genes involved in inflammatory and immune responses, *GLIPR1*, *EEA1*, *TCL1A, TCL1B*, *UBA7*, *RNF123*, *MST1* and *RHOA,* were also found*. GLIPR1*, *UBA7, RNF123* and *EEA1* participate in several immune system-related pathways. T-cell leukemia/lymphoma 1A and 1B (*TCL1A* and *TCL1B*) genes are involved in T- and B-cell development [64]. The macrophage stimulating 1 (*MST1*) gene regulates macrophage activity during inflammation [65, 66]. Ras homolog family member A (*RHOA*) is a small GTPases of the Rho family, which regulates several cellular processes including actin cytoskeleton reorganization and inflammation [67, 68].

Four other potential candidate genes for uniformity were identified. Leucine zipper putative tumor suppressor 1 (*LZTS1*), phospholipid phosphatase related 4 and 5 (*PLPPR4* and *PLPPR5)* are involved in neuronal plasticity [69, 70], and bone morphogenetic protein receptor type 1A (*BMPR1A*) playing an important role in bone formation, which was recently found associated with body size in sheep [71]. Neuronal plasticity is the ability of the nervous system to adapt to environmental changes, which denotes another potential class of genes determining uniformity.

In the common regions between ln_${\upsigma}_{\widehat{\mathrm{e}}}^2$ and dEBV_v_r0_, genes related to metabolism (*NUDT9*, *SPARCL1* and *MAN2B2)* and mineralization of tissues like bones and teeth (*DMP1* and *DSPP* [72]), are likely to be linked to uniformity on Chr6_105. It is not unusual to find common genes related to stature, height and body weight in beef cattle (e.g. [14, 73–75]). Such findings highlight the strong relationship between height and weight, partially explained by common regulatory mechanisms.

Additionally, both common regions (Chr6_105 and 9_8) contain genes with neuronal functions: *PPP2R2C, JAKMIP1* and *ADGRB3*. Protein phosphatase 2, regulatory subunit B, gamma (*PPP2R2C)* and adhesion G protein-coupled receptor B3 (*ADGRB3)* were previously related to learning and memory (synaptic plasticity) and synaptogenesis in mice [76, 77]*.* Janus kinase and microtubule-interacting protein 1 (*JAKMIP1)* was associated with social behavior in mice through its function on neuronal translation and was related to T cell-mediated cytotoxicity [78, 79]. These results together with dEBV_v_ and ln_${\upsigma}_{\widehat{\mathrm{e}}}^2$ provide evidence that the nervous system is also modulated according to environmental perturbations. Previously, studies reporting such neuronal plasticity in response to changes in nutrition and temperature were observed in *Drosophila* [80–82]. Therefore, together with other tissues, the nervous system seems to be a potential modulator of the mechanisms underlying uniformity.

### Response variables

The high correlation between dEBV_m_ and dEBV_v_ resulted in 8 common regions affecting simultaneously mean and residual variance. Such findings are in line with Wolc et al. [7] who found correlations ranging from 0.54 to 0.74 and a region explaining a large proportion of the genetic variance of the mean and standard deviation (SD) of egg weight at different ages. The authors noted that it was not a simple scale effect, because the effect of the QTL on mean egg weight (measured between 26 and 28 weeks of age) was about 4% of the mean egg weight and on SD egg weight at the same age was about 5% of the mean SD. More recently, Sell-Kubiak et al. [13] also reported a high genetic correlation, 0.49, between litter size and its variation, but no overlapped regions were found.

The dEBV_v_ not only captured common effects with the mean of YW but also its residual variance by sharing regions (among top 20 by variance explained) with dEBV_v_r0_ and ln_${\upsigma}_{\widehat{\mathrm{e}}}^2$, Chr12_5 and Chr22_52, respectively. These results suggest that the mean and uniformity of YW are partially under different genetic control. Though some of the genes found on chromosome 22 and most of those in common between dEBV_m_ and dEBV_v_ are involved in metabolism (e.g. *PLAG1, DPYD*, *AK7, GMPPB, AMT* and *GLUD1*), a process most probably related to both, mean and uniformity.

The weak correlations among dEBV_m_ and the response variables that assumed null r_mv_ emphasize the role of the latter to find genomic regions that are specifically affecting residual variance and not the mean of YW. Generally, data transformation is used as the main tool to reduce mean-variance relationship in studies on uniformity (e.g. [83–85]). This is the first study on uniformity reporting null r_mv_ as a strategy to deal with scale effects and showing the differences in terms of associated regions when comparing to GWAS using dEBV_v_ when non-null r_mv_ was assumed.

Knowing the importance of considering potential scale effects and confounding between mean and residual variance, the next step is to discuss the suitability of the different response variables to be used in a GWAS for uniformity. Based on our results, we recommend considering the following aspects regarding the response variable and data structure. Firstly, we need to take into account the nature of the trait because small genetic variance and low heritabilities are often found for uniformity. Previously we estimated a heritability for residual variance of YW even smaller (0.007; [19]) than has been reported for similar traits in other livestock populations [86]. Probably this also may have contributed to the small (or none) number of SNPs that reached the significance threshold (BF > 20) when we analyzed the response variables for residual variance. In this context, irrespective of the response variable, a larger sample size is required to increase the power of GWAS [87].

Secondly, we used two different procedures to obtain response variables to perform GWAS for uniformity. The first one was the deregressed EBV; deregression, which removes both the contribution of parents and the shrinkage present in the EBV. On the other hand, the second one resulted in a measure, ln_${\upsigma}_{\widehat{\mathrm{e}}}^2$, with less adjustments compared to deregression. However, some non-genetic effects, such as the contemporary group (CG) effect on the variance may have affected the within-family variance estimate ln_${\upsigma}_{\widehat{\mathrm{e}}}^2$, while they are accounted for when we used dEBV since non-genetic effects, were fitted in the DHGLM in the residual variance part of the model. In addition, the assumptions of null and non-null r_mv_ were assumed to measure the influence of the mean and/or potential scale effects on the residual variance. Therefore, analyzing each response variable, we can conclude that: i) dEBV_v_ was greatly affected by the mean; ii) dEBV_v_r0_ had low accuracy by assuming null r_mv,_ which requires a large amount of phenotypic information and/or genotyped sires; and iii) ln_${\upsigma}_{\widehat{\mathrm{e}}}^2$ was less related to the mean and less regressed compared to dEBV, but it may include some non-genetic effects, like CG. It is clear that all three approaches have advantages and disadvantages. Given the fact that ln_${\upsigma}_{\widehat{\mathrm{e}}}^2$ is likely more independent to the mean than dEBV_v_, there may be in this case a small preference for this response variable as it behaved also better in the Markov chain Monte Carlo (MCMC). Furthermore, considering different response variables can be beneficial, windows that appear in multiple approaches may have a higher credibility and may help in understanding the role of genes affecting the mean, the residual variance or both and whether genes controlling the variance beyond scale effects.

## Conclusions

Using solutions from a DHGLM that assumed null genetic correlation between mean and residual variance was a suitable strategy to identify genomic regions affecting uniformity less dependent on the mean. Thirteen and four SNPs were associated with dEBV_v_r0_ and ln_${\upsigma}_{\widehat{\mathrm{e}}}^2$, respectively, and additional common regions were observed among the responses variables. In all these regions, promising biological candidate genes for uniformity of YW were related not only with metabolic genes, but also stress, inflammatory and immune responses, mineralization, neuronal activity and bone formation. No strong evidence was found in favor of using a specific response variable for GWAS, while using different response variables was beneficial from the biological point of view, since more evidence on candidate regions can be found.

## Methods

The phenotypic data used in this study are described in Iung et al. [19]. Here, genotypic information from 423 influential Nellore sires from the Alliance Nellore database with a large number of evaluated progeny were available. The number of progeny per sire considered for the study ranged from 50 to 10,180, with an average of 411 (see distribution of progeny per sire in Additional file 2). In total, 194,628 yearling weight measurements from the progeny were used to estimate the response variables used in the GWAS.

### Response variables in the association studies

All responses variables used solutions or residuals from DHGLM fitted in our previous study [19]. The DHGLM is an iterative approach that estimates simultaneously genetic parameters for the mean and residual variance. In our previous study a sire DHGLM was used as follows: $$ \left[\begin{array}{c}\mathbf{y}\\ {}\boldsymbol{\uppsi} \end{array}\right]=\left[\begin{array}{cc}\mathbf{X}& \mathbf{0}\\ {}\mathbf{0}& {\mathbf{X}}_{\mathbf{v}}\end{array}\right]\left[\begin{array}{c}\mathbf{b}\\ {}{\mathbf{b}}_{\mathbf{v}}\end{array}\right]+\left[\begin{array}{cc}\mathbf{Z}& \mathbf{0}\\ {}\mathbf{0}& {\mathbf{Z}}_{\mathbf{v}}\end{array}\right]\left[\begin{array}{c}\mathbf{s}\\ {}{\mathbf{s}}_{\mathbf{v}}\end{array}\right]+\left[\begin{array}{c}\mathbf{e}\\ {}{\mathbf{e}}_{\mathbf{v}}\end{array}\right] $$, where **y** and **ψ** are vectors of response variables for the mean and residual variance models (denoted with the subscript v), respectively, **b** and **b**_**v**_ are vectors of fixed effects and covariates (contemporary group, linear and quadratic effects of animal age, nested within sex), **s** and **s**_**v**_ are vectors of random sire genetic effects, **e** and **e**_**v**_ are vectors of random residual effects, **X, X**_**v**_**, Z** and **Z**_**v**_ are corresponding incidence matrices. This approach was chosen because of its better predictability compared to a two-step approach, as previously stated by Iung et al. [19]. Mean of YW was evaluated through dEBV_m_ obtained following the deregression procedure proposed by Garrick et al. [88] and using EBV of the mean from DHGLM [12]. For the residual variance of YW, different response variables were analyzed: i) dEBV_v_: deregressed EBV of the residual variance from a DHGLM assuming non-null genetic correlation between mean and residual variance (r_mv_ ≠ 0); ii) dEBV_v_r0_: deregressed EBV of the residual variance from a DHGLM assuming r_mv =_ 0; and iii) ln_${\upsigma}_{\widehat{\mathrm{e}}}^2$, log-transformed variance of estimated residuals for each sire family, using residuals from the mean model of a DHGLM also assuming r_mv =_ 0. The log-transformation was used to reduce mean-variance relationship and correct for non-normality of the residual variances. Fitting the DHGLM assuming null r_mv_ was intended to reduce the impact of the mean of YW may have on the EBV in the residual variance part of the model, given the strong estimate of r_mv_ on YW previously reported for this population [19]. For instance, the EBV_v_ and EBV_m_ would be even higher correlated than the r_mv_ because of using the information on mean YW for both sets of EBV. When using null r_mv_, it is expected to have a higher possibility to find regions only affecting the residual variance. Descriptive statistics of each response variable are presented in Table 6 and the distribution of them in Additional file 2.

**Table 6:** Descriptive statistics for mean and residual variance of yearling weight

Phenotypes	N	Mean (SD)	Minimum	Maximum
Mean				
dEBV_m_	423	6.084 (15.109)	−67.120	45.790
Uniformity				
dEBV_v_	423	0.07274 (0.248)	−0.920	0.775
dEBV_v_r0_	423	2.55E-05 (0.442)	−1.403	1.494
ln_${\upsigma}_{\widehat{\mathrm{e}}}^2$	423	6.313 (0.273)	5.505	7.295

dEBV_m_ and dEBV_v_: deregressed EBV for mean and residual variance of yearling weight, respectively; dEBV_v_r0_ and ln_${\upsigma}_{\widehat{\mathrm{e}}}^2$: deregressed EBV for residual variance and log-transformed variance of estimated residuals, respectively, both assuming null genetic correlation between mean and residual variance

*N* number of observations, *SD* standard deviation

### Genotypes

Genotypic information from 423 Nellore bulls was used, being 415 genotyped with the Illumina® BovineHD chip (HD) and 8 with Illumina BovineSNP50 BeadChip (Illumina Inc., San Diego, CA, USA) and then imputed to HD (777 k) using FImpute software [89]. In the quality control of genotypes, SNPs located on the sex chromosomes, with minor allele frequency (MAF) lower than 0.02, *p*-value for Hardy-Weinberg equilibrium test (HWE) less than 10^− 5^ and highly correlated SNPs (r^2^ > 0.99) within a window of 100 consecutive SNPs were removed, so that 333,877 SNPs remained for analysis. All genotyped bulls had a call rate higher than 0.90, passing the quality control.

### GWAS

The SNP effects were estimated using Bayes C method [90], a mixture model which assumes that there is a smaller fraction of SNPs (1-π) with large effects and a large proportion (π) with zero or near zero effects on the trait, as follows:

$$ \mathrm{y}=1\upmu +\sum \limits_{\mathrm{i}=1}^{\mathrm{N}}{\mathrm{z}}_{\mathrm{i}}{\mathrm{a}}_{\mathrm{i}}{\updelta}_{\mathrm{i}}+\mathrm{e} $$

where **y** is the vector of response variables (dEBV_m_, dEBV_v_, dEBV_v_r0_ and ln_${\upsigma}_{\widehat{\mathrm{e}}}^2$), μ is the overall mean, **1** is a vector of ones, **z**_**i**_ is the vector of genotypes of the animals for the i^th^ SNP, **a**_**i**_ is the allele substitution effect of the i^th^ SNP, **δ**_**i**_ is an indicator variable set to 1 if the i^th^ SNP has an non-zero effect on the trait and to 0 otherwise, **e** is the vector of random residual effects and N is the number of SNPs. It was assumed **a**_**i**_ ~ N(**0**,${\upsigma}_{\mathrm{a}}^2$) and **e** ~ N(0, **R**${\upsigma}_{\mathrm{e}}^2$), where ${\upsigma}_{\mathrm{a}}^2$ is the variance of SNP effects, ${\upsigma}_{\mathrm{e}}^2$ is the residual variance and **R** is a diagonal matrix whose elements account for heterogeneous residual variance across observations. When dEBV was the response variable, the diagonal elements of **R** were derived following Garrick et al. [88], whereas when ln_${\upsigma}_{\widehat{\mathrm{e}}}^2$ was the response, such elements were equal to the reciprocal of the number of progeny of each sire. The **R**-accounted for differences in sampling variance due to different numbers of progeny per sire, even observing low correlations between the number of progeny per sire and each response variable (0.021 to 0.145). In addition, scaled inverse chi-squared prior distributions with *v* degrees of freedom and scale parameter S were assumed for ${\upsigma}_{\mathrm{a}}^2$ and ${\upsigma}_{\mathrm{e}}^2$. In our study, π was fixed at 0.999, which means that 0.1% of the SNPs fitted in the model, i.e. about 334, have an effect on the trait. Such a criterion was assumed aiming to obtain stronger signals of candidate QTL and also due to the limiting number of genotypes.

The analyses were performed using the GS3 software [91]. Chains of 550,000 and 250,000 iterations, after discarding the first 150,000 and 50,000 as burn-in, were generated for analyses pertaining to the three response variables for residual variance and mean of YW, respectively, saving samples every 50 iterations in both cases. Convergence was assessed through Geweke test [92] using CODA R package [93].

The significance of each association was evaluated through the Bayes Factor (BF):

$$ \mathrm{BF}=\frac{\left(\frac{\mathrm{p}}{1-\mathrm{p}}\right)}{\left(\frac{\uppi}{1-\uppi}\right)} $$

where p and π are the posterior and prior probability of a SNP being included in the model, i.e. having a non-zero effect, respectively. Suggestive and strong evidences were assigned to SNPs with BF greater than 3 and 20, respectively [94, 95]. In addition, the proportion of genetic variance explained by SNPs within non-overlapping 1-Mb windows were calculated. A window size of 1-Mb was chosen since SNPs within this distance present moderate to high (> 0.2) pairwise linkage disequilibrium (LD; measured by r^2^) (see Additional file 3). In total, SNPs were allocated to 2522 windows, with an average (SD) of 132 (42) SNPs. The top 20 windows that explained the largest proportion of genetic variance were compared between responses variables.

### Functional annotation

The shared 1-Mb windows (among the top 20 that explained the largest proportion of genetic variance) between response variables and windows with SNPs that showed a strong association (BF > 20) were screened to identify potential candidate genes using Ensembl Genome Browser (http://www.ensembl.org), UMD3.1 bovine genome assembly [96]. Previously reported QTL regions overlapping such windows were identified from Cattle QTLdb [97]. Functional enrichment based on Gene Ontology (GO) terms and pathway analysis using Blast2GO PRO and Reactome [98, 99], respectively, was performed on these regions in order to help us to determine potential candidate genes for uniformity. Only the promising candidate genes, according to their function, pathway or related biological process, were discussed here (the complete gene list and annotations are shown in Additional file 1). The LD maps within each 1-Mb window discussed here were obtained using the R package LDheatmap [100] (Additional file 3).

## Additional files

Additional file 1: Complete list of the genes located within 1-Mb windows harboring SNPs with strong association by trait. Gene Ontology (GO) terms and pathways of each gene were obtained using Blast2GO and Reactome. (XLSX 99 kb)

Additional file 2: Distribution of number of progeny per sire and responses variables. (DOCX 242 kb)

Additional file 3: Manhattan plot and linkage disequilibrium (LD) map in each 1-Mb window discussed in our study. (PDF 419 kb)

## Abbreviations

BF: Bayes factor
Chr: Chromosome
dEBV: Deregressed EBV
DHGLM: Double hierarchical generalized linear model
EBV: Estimated breeding value
GWAS: Genome-wide association study
HWE: Hardy-Weinberg equilibrium
ln_${\upsigma}_{\widehat{\mathrm{e}}}^2$: log-transformed variance of estimated residuals
MAF: Minor allele frequency
Mb: Mega bases
QTL: Quantitative trait loci
SNP: Single nucleotide polymorphism
YW: Yearling weight
