# Supplementary material for: Genomic regions underlying uniformity of yearling weight in Nellore cattle evaluated under different response variables
Source: BMC Genomics. 2018 Aug 16;19:619. doi: 10.1186/s12864-018-5003-4 (PMC6097312; doi:10.1186/s12864-018-5003-4)
Supplement: Supplementary file 2 — Distribution of number of progeny per sire and responses variables. (DOCX 242 kb) [file 12864_2018_5003_MOESM2_ESM.docx]

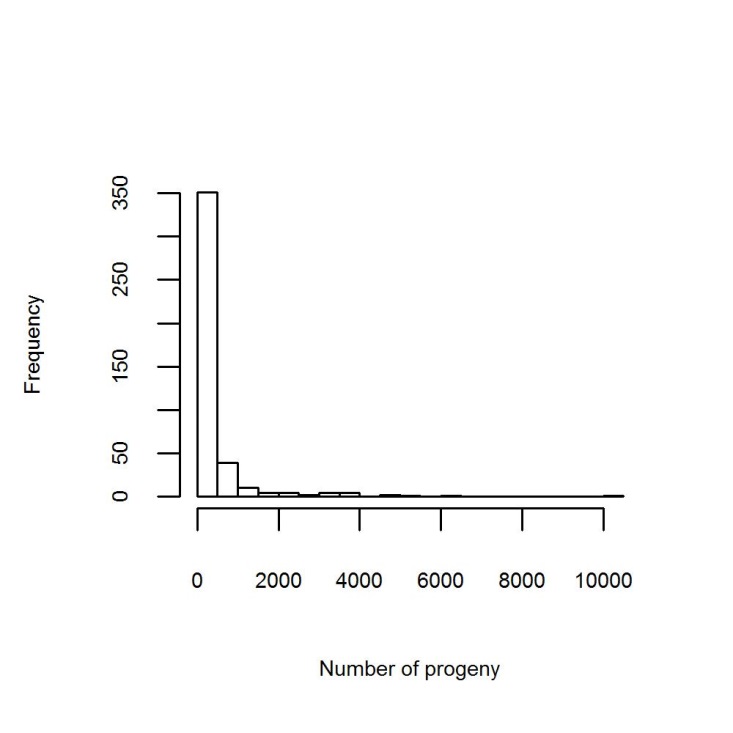


Figure 3a. Distribution of number of progeny per sire.

| 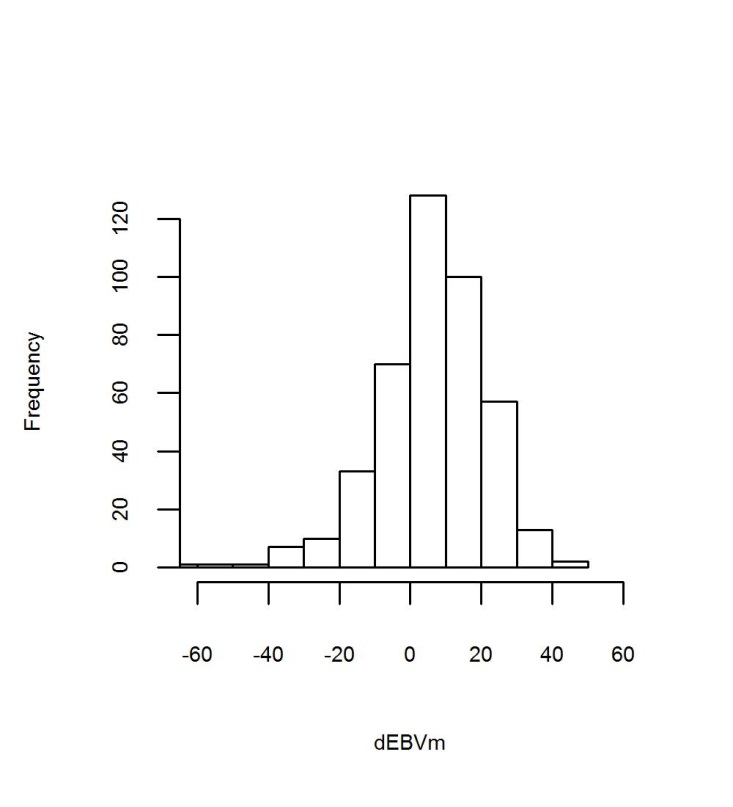 | 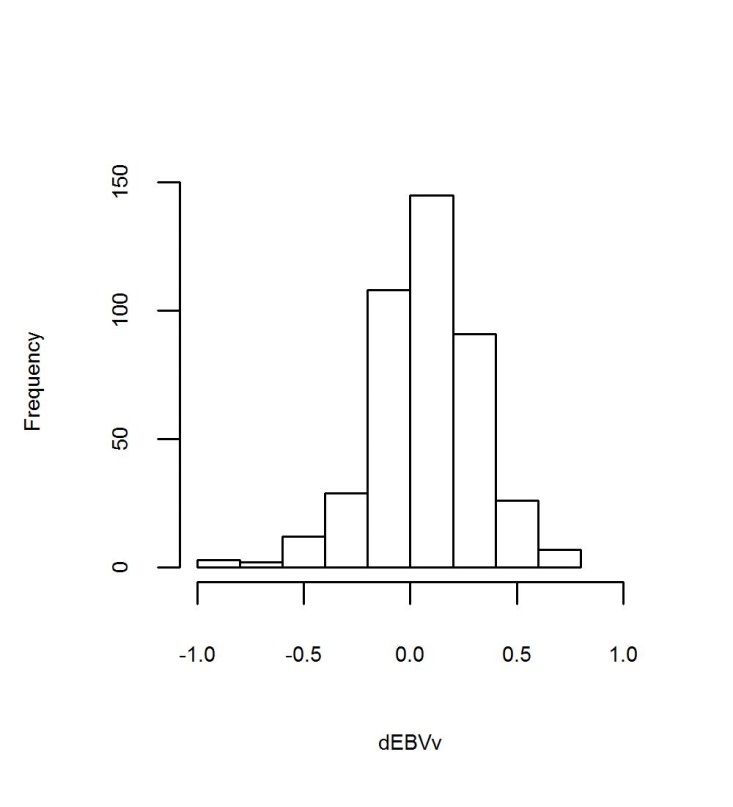 |
| --- | --- |
| 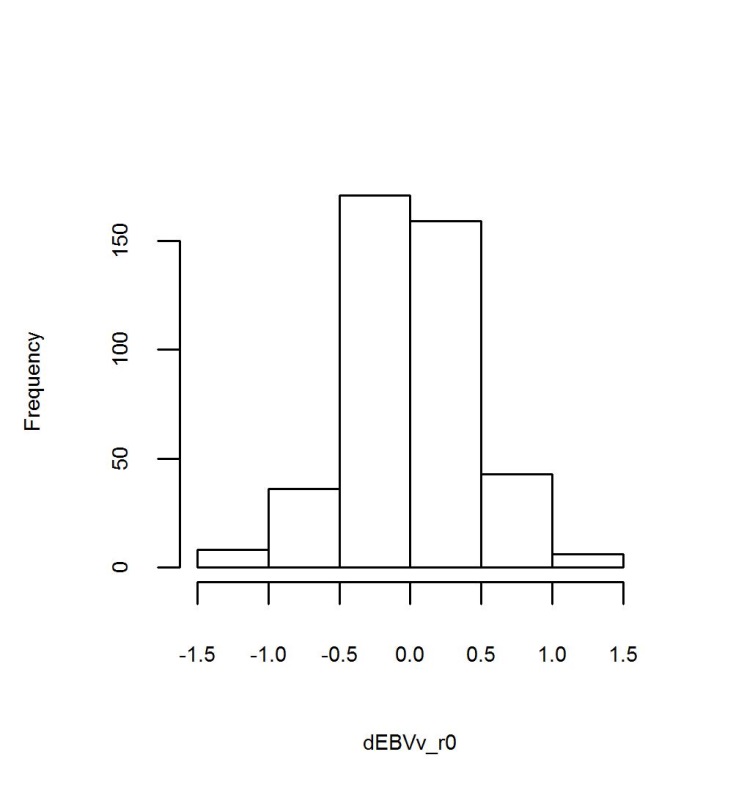 | 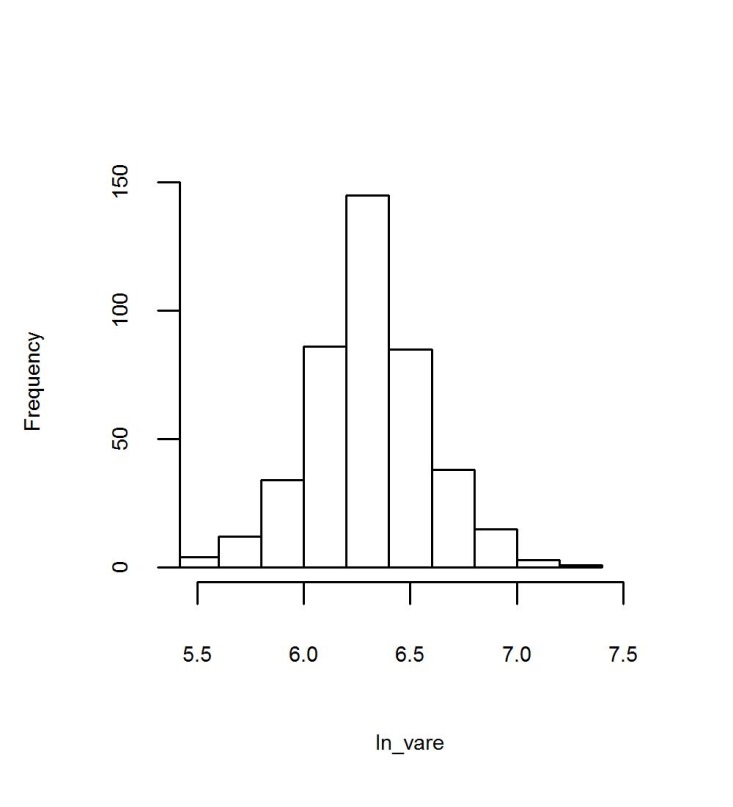 |

Figure 3b. Distribution of the four responses variables (dEBV_m,_ dEBV_v,_ dEBV_v_r0_ and ln_$\text{σ}_{\text{ê}}^{\text{2}}$) used in this study.
