# Supplementary material for: Genomic regions underlying uniformity of yearling weight in Nellore cattle evaluated under different response variables
Source: BMC Genomics. 2018 Aug 16;19:619. doi: 10.1186/s12864-018-5003-4 (PMC6097312; doi:10.1186/s12864-018-5003-4)
Supplement: Supplementary file 3 — Manhattan plot and linkage disequilibrium (LD) map in each 1-Mb window discussed in our study. (PDF 419 kb) [file 12864_2018_5003_MOESM3_ESM.pdf]

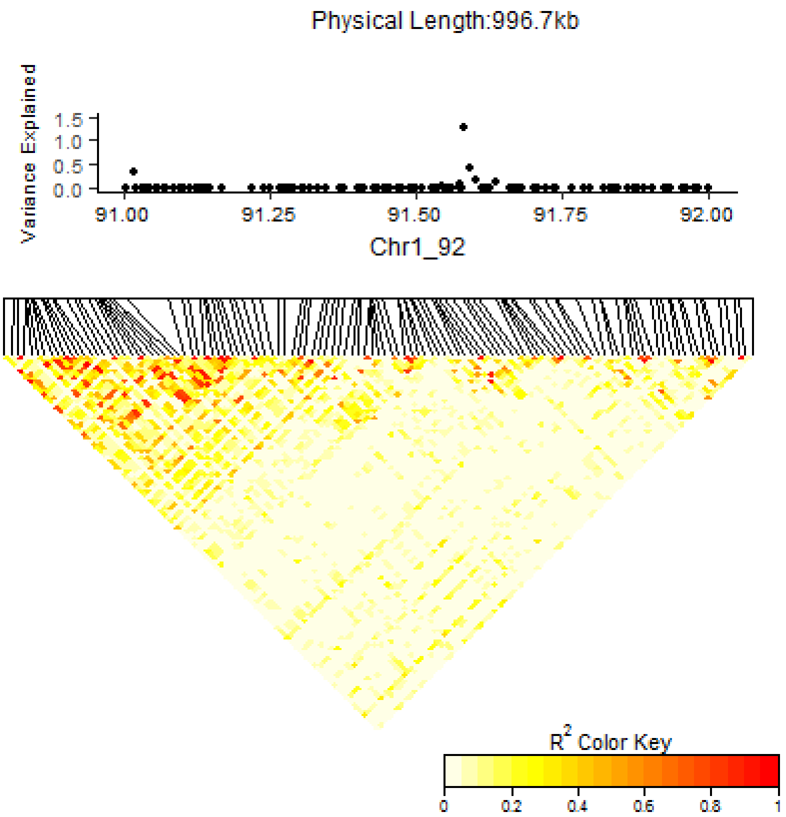

Figure 4a. Manhattan plot of the variance explained by each SNP in the window Chr1_92 for $dEBV_m$ and linkage disequilibrium (LD; r^2)

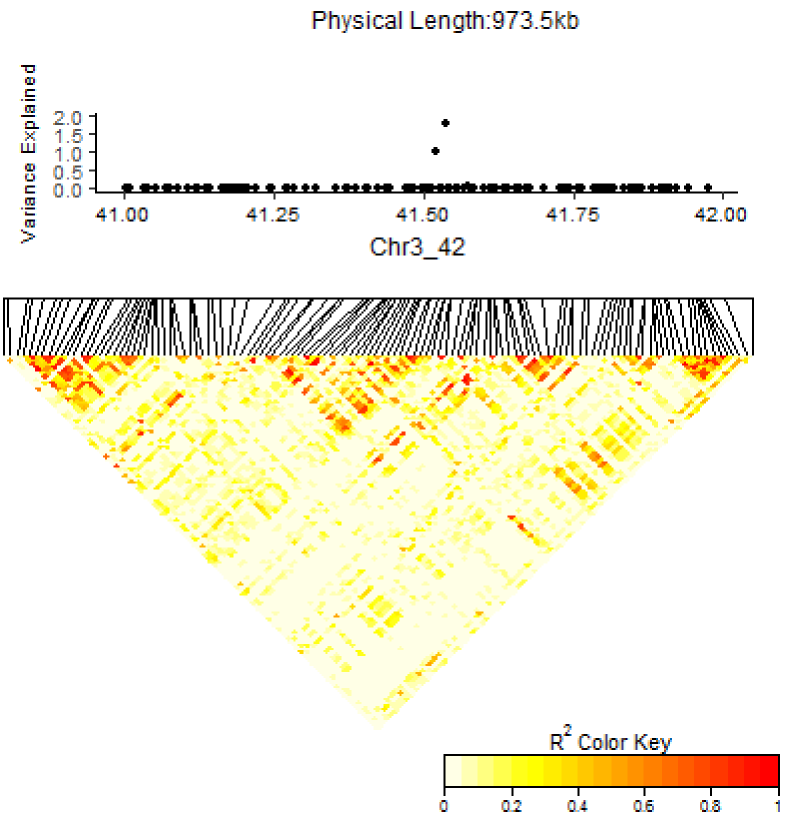

Figure 4b. Manhattan plot of the variance explained by each SNP in the window Chr3_42 for $dEBV_m$ and linkage disequilibrium (LD; r^2)

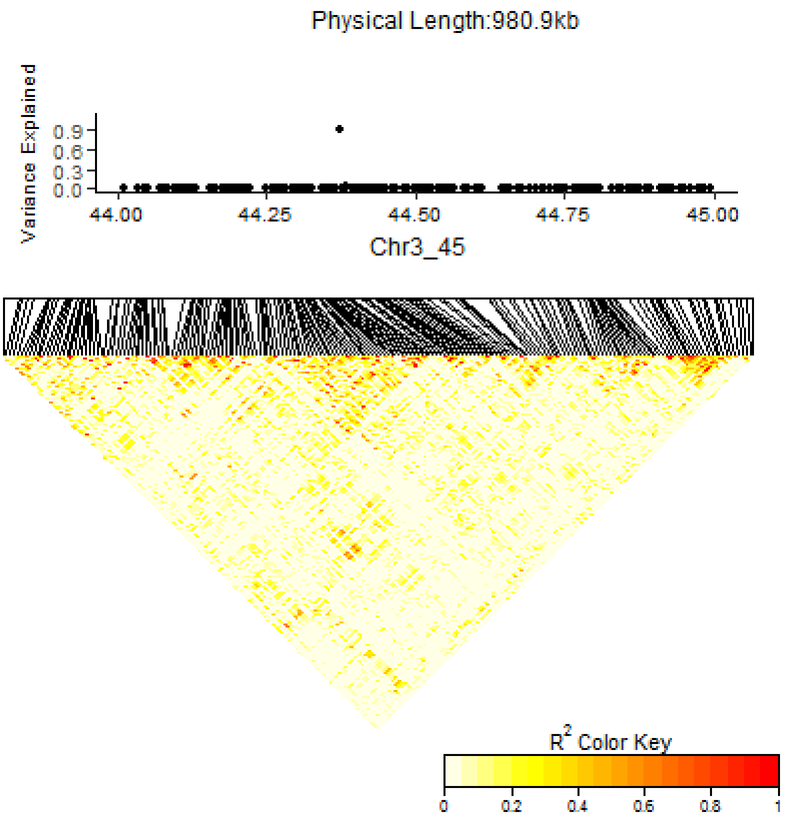

Figure 4c. Manhattan plot of the variance explained by each SNP in the window Chr3_45 for $dEBV_m$ and linkage disequilibrium (LD; r^2)

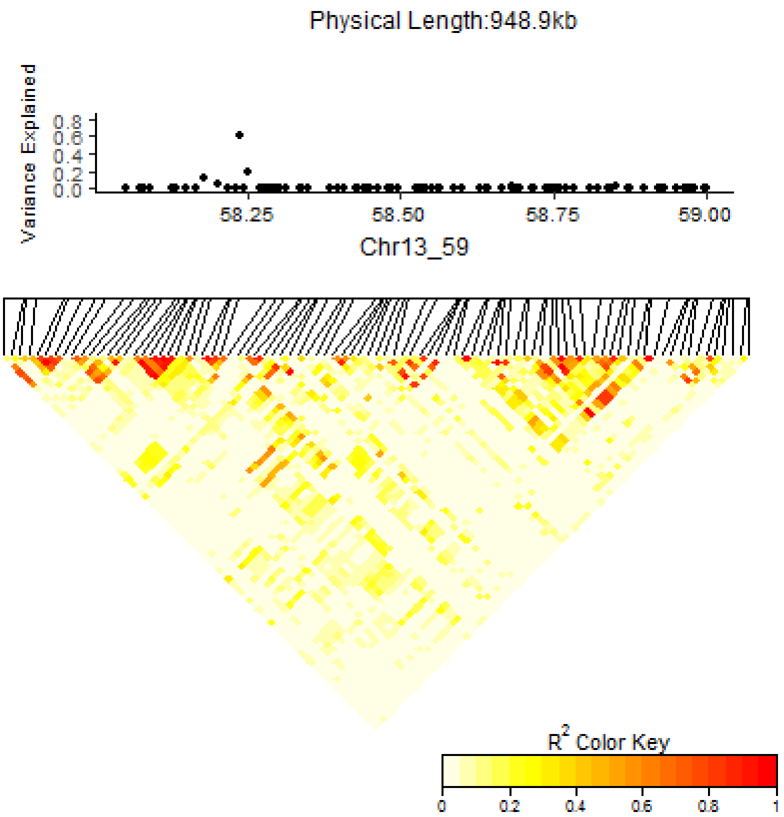

Figure 4d. Manhattan plot of the variance explained by each SNP in the window Chr13_59 for $dEBV_m$ and linkage disequilibrium (LD; r^2)

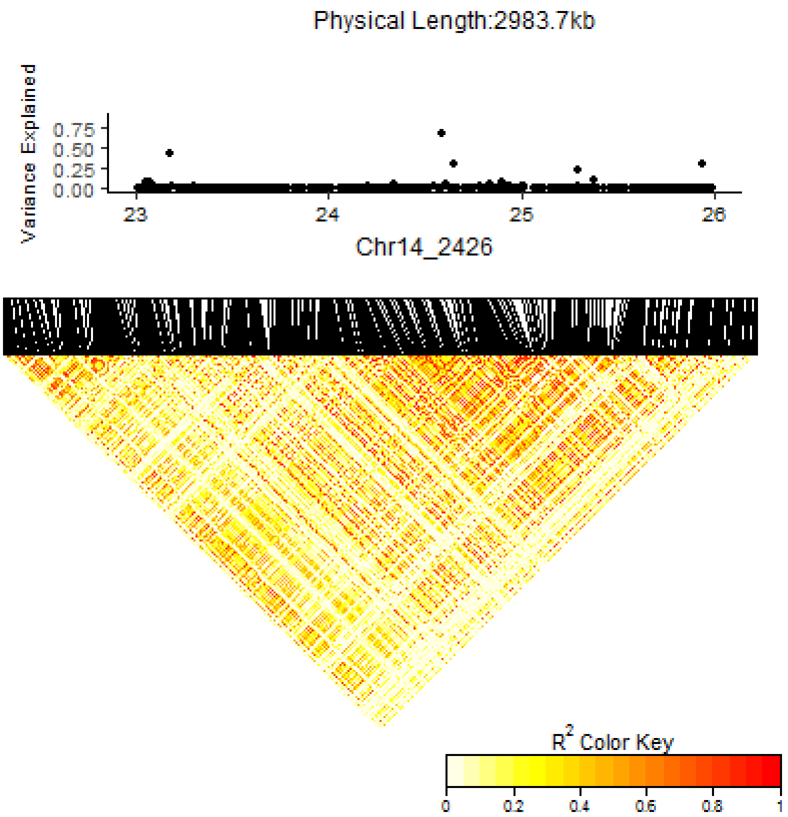

Figure 4e. Manhattan plot of the variance explained by each SNP in the window Chr14_24:26 for $dEBV_m$ and linkage disequilibrium (LD; r^2)

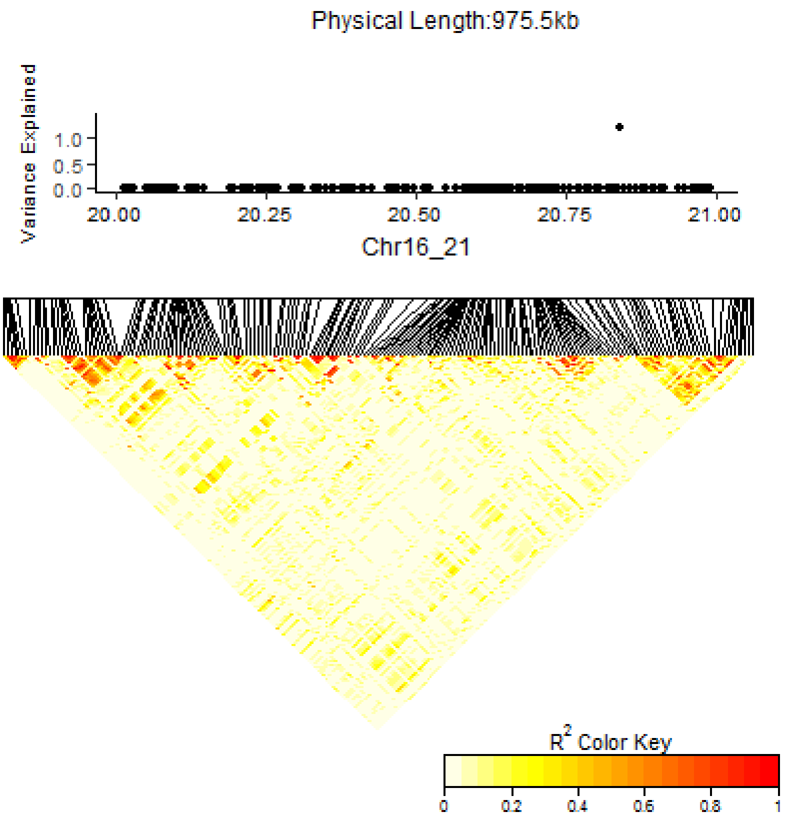

Figure 4f. Manhattan plot of the variance explained by each SNP in the window Chr16_21 for $dEBV_m$ and linkage disequilibrium (LD; r^2)

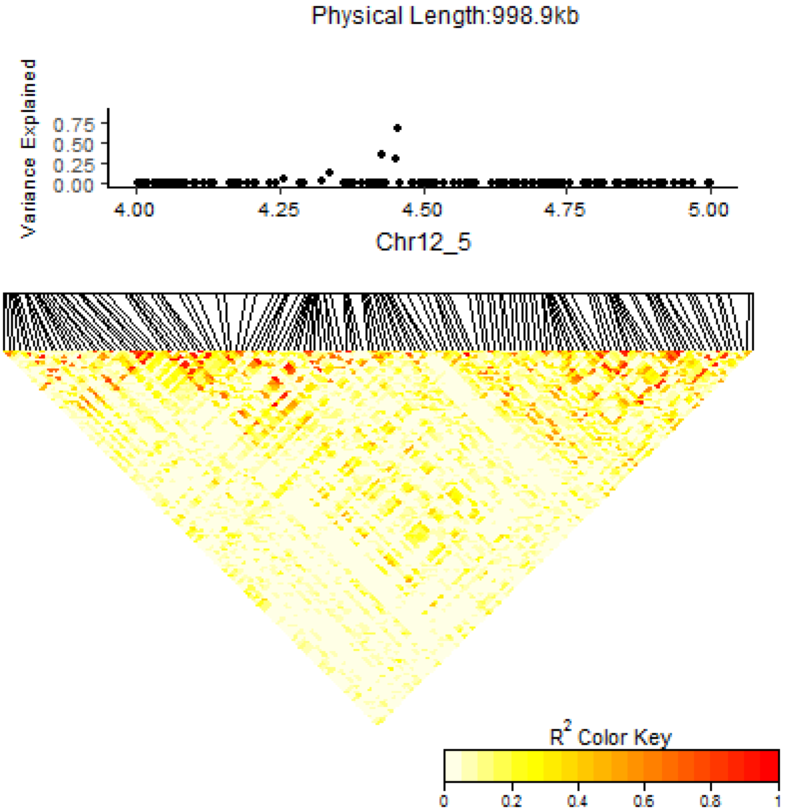

Figure 4g. Manhattan plot of the variance explained by each SNP in the window Chr12_5 for $dEBV_v$ and linkage disequilibrium (LD; r^2)

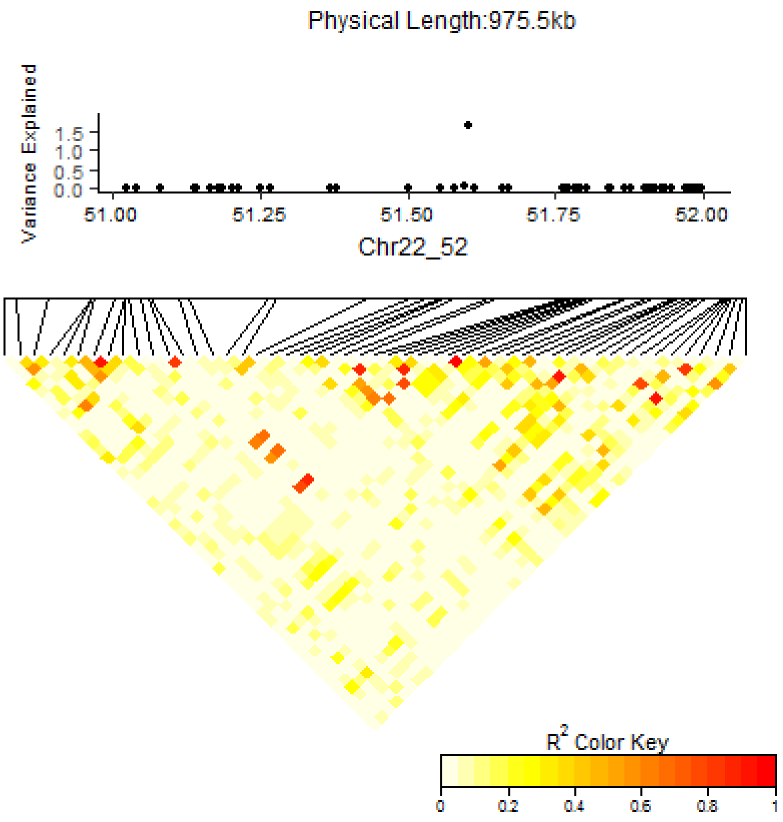

Figure 4h. Manhattan plot of the variance explained by each SNP in the window Chr22_52 for $dEBV_v$ and linkage disequilibrium (LD; r^2)

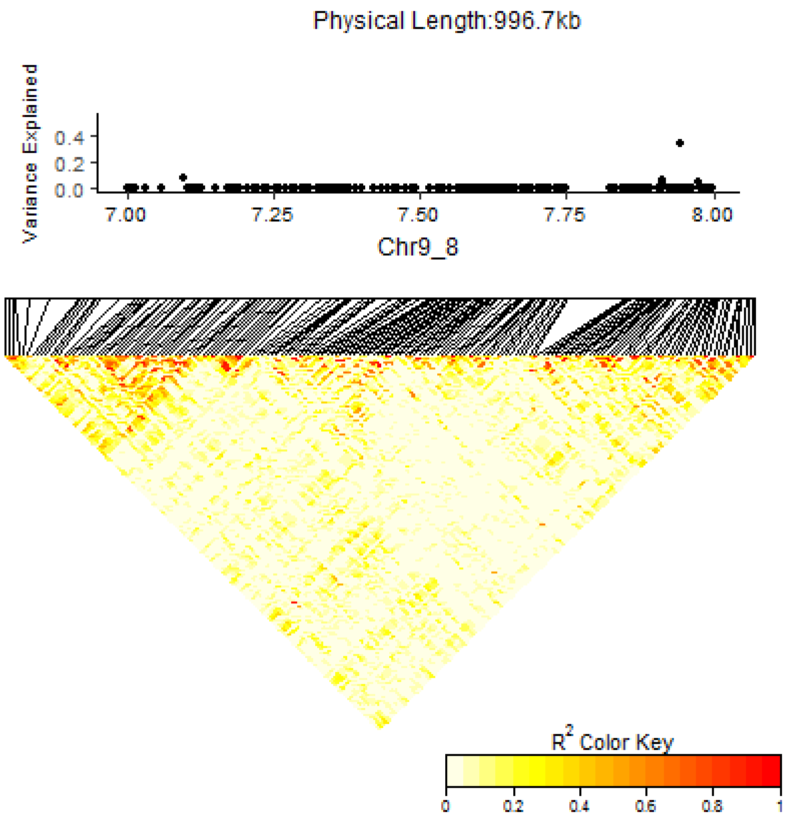

Figure 4i. Manhattan plot of the variance explained by each SNP in the window Chr9_8 for ln_$\sigma^2_{\hat{e}}$ and linkage disequilibrium (LD; r^2)

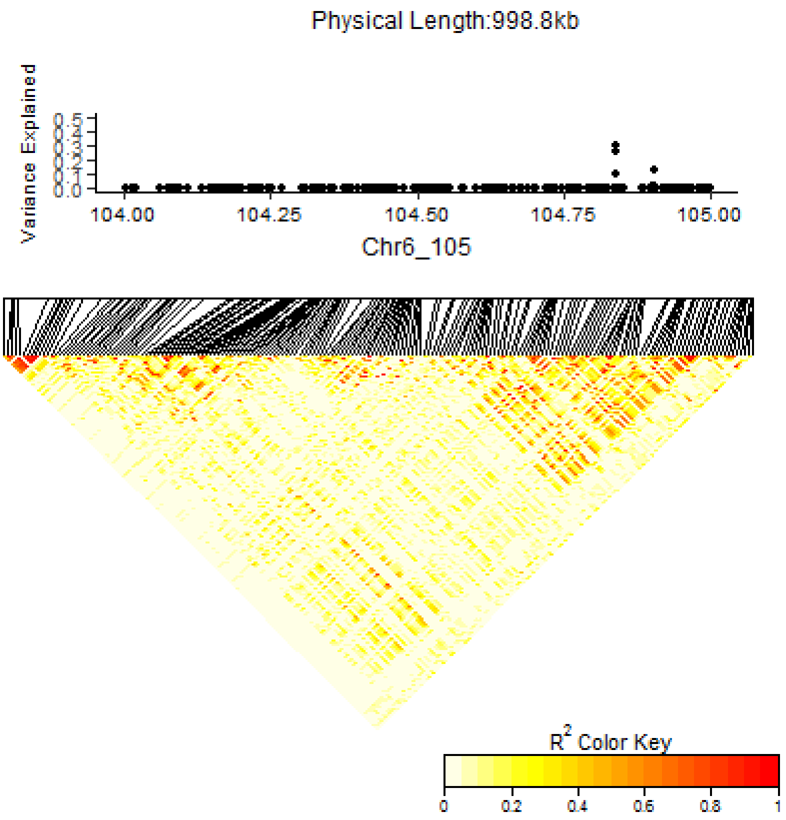

Figure 4j. Manhattan plot of the variance explained by each SNP in the window Chr6_105 for ln_$\sigma^2_{\hat{e}}$ and linkage disequilibrium (LD; r^2)

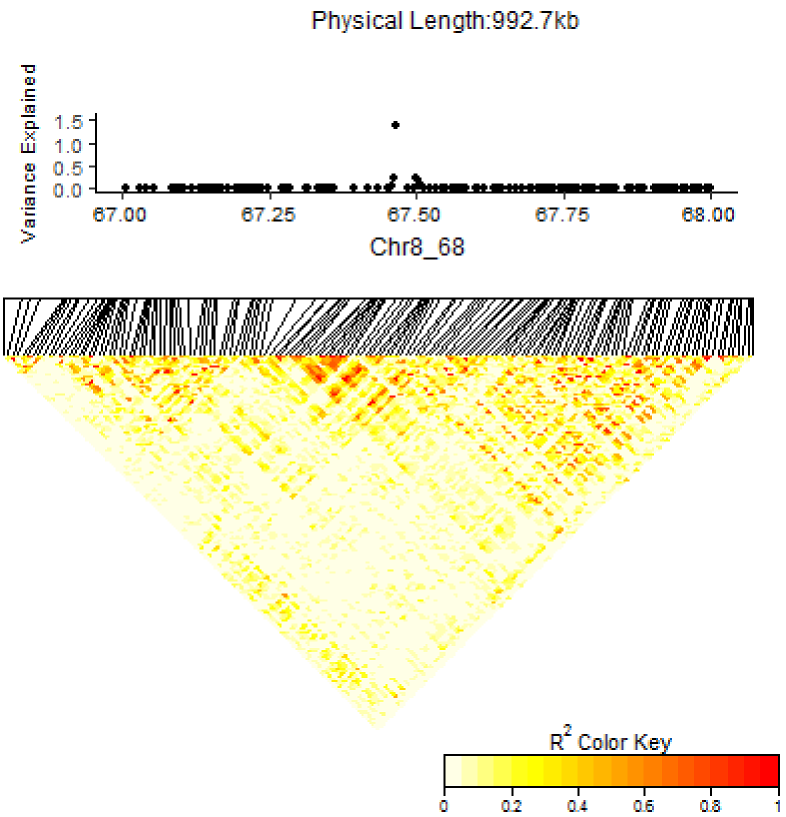

Figure 4k. Manhattan plot of the variance explained by each SNP in the window Chr8_68 for $\ln\_\sigma^2_{\hat{e}}$ and linkage disequilibrium (LD; r^2)

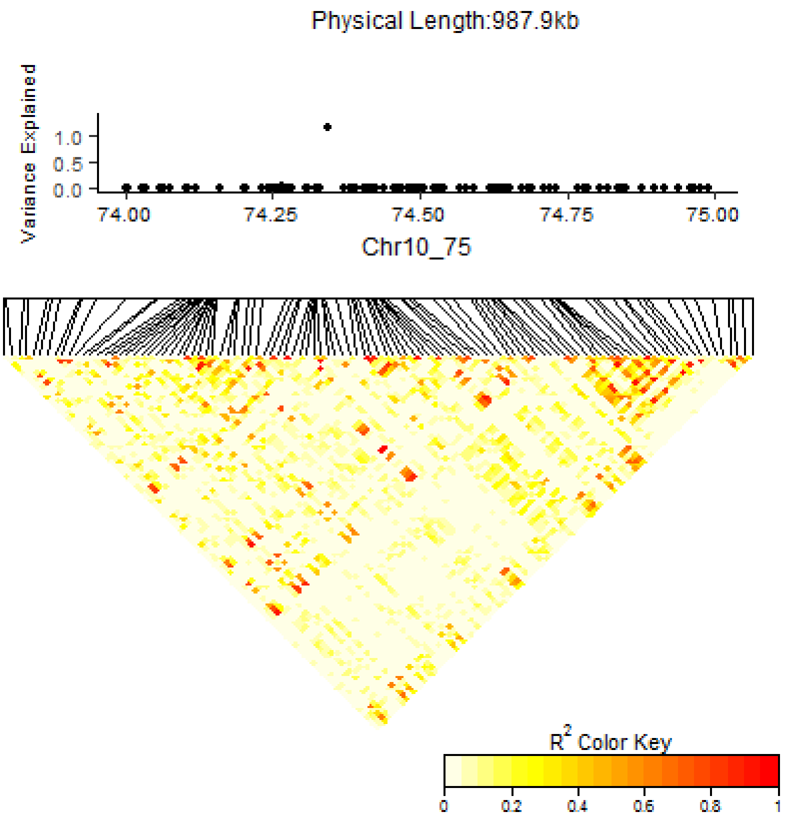

Figure 4l. Manhattan plot of the variance explained by each SNP in the window Chr10_75 for ln_$\sigma^2_{\hat{e}}$ and linkage disequilibrium (LD; r^2)

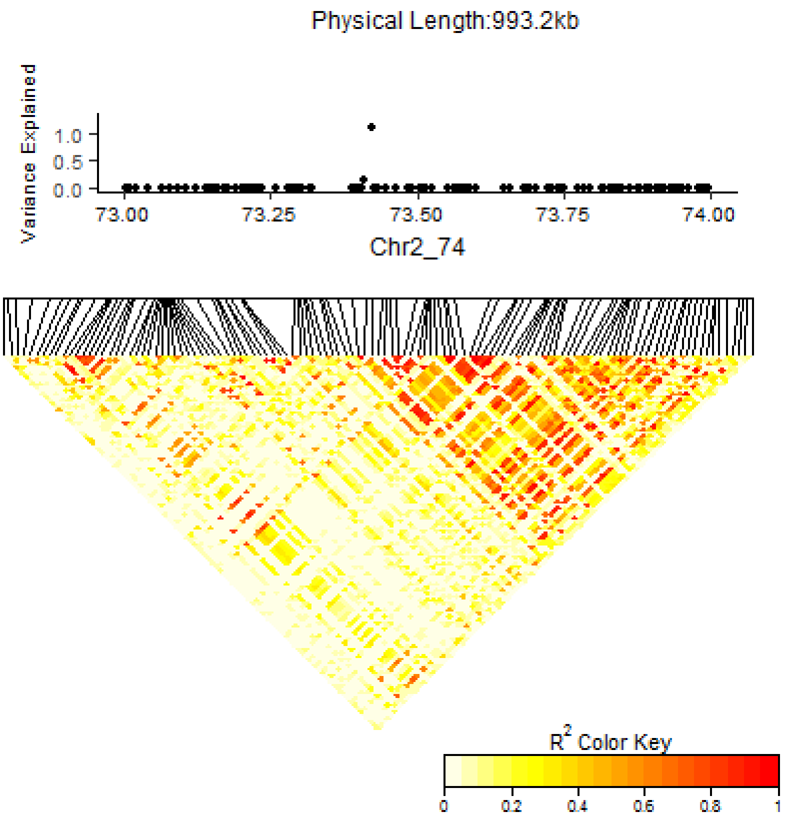

Figure 4m. Manhattan plot of the variance explained by each SNP in the window Chr2_74 for ln_$\sigma^2_{\hat{\varepsilon}}$ and linkage disequilibrium (LD; r^2)

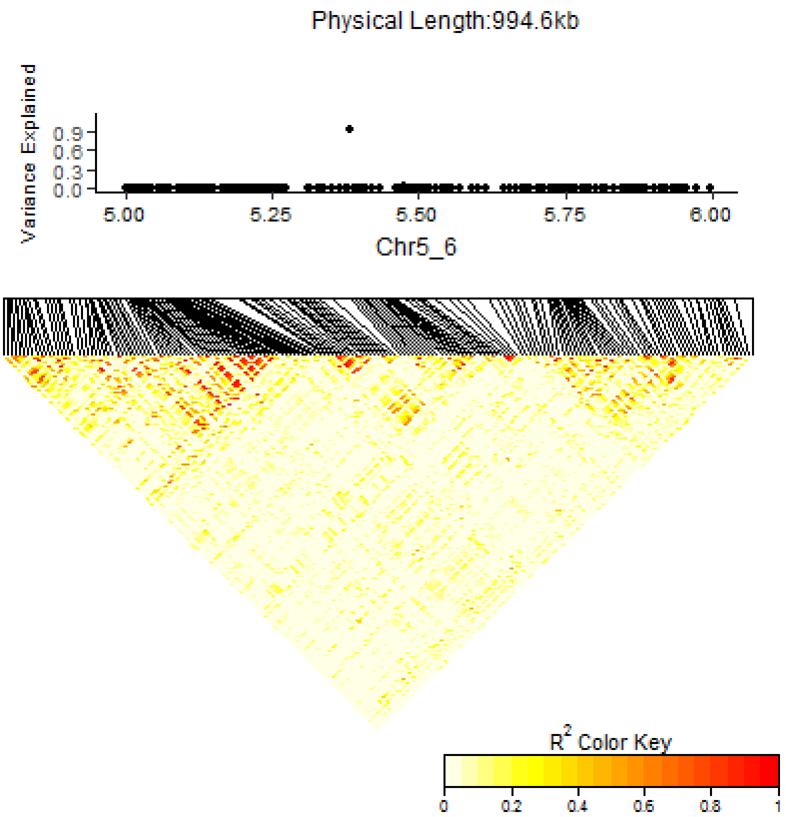

Figure 4n. Manhattan plot of the variance explained by each SNP in the window Chr5_6 for ln_$\sigma^2_{\hat{e}}$ and linkage disequilibrium (LD; r^2)

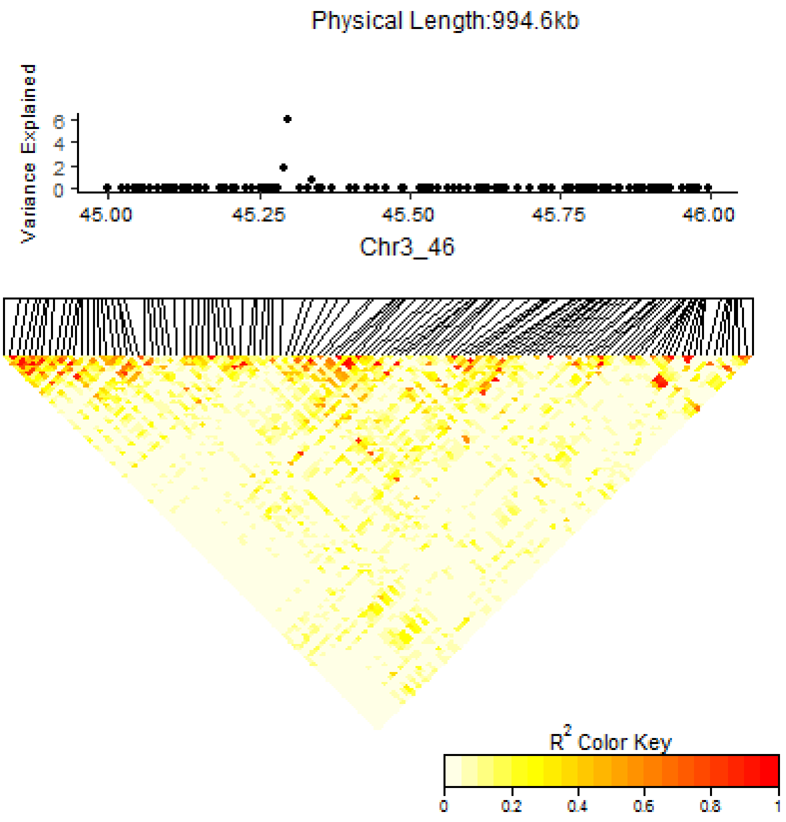

Figure 4p. Manhattan plot of the variance explained by each SNP in the window Chr3_46 for $dEBV_v$ and linkage disequilibrium (LD; r^2)

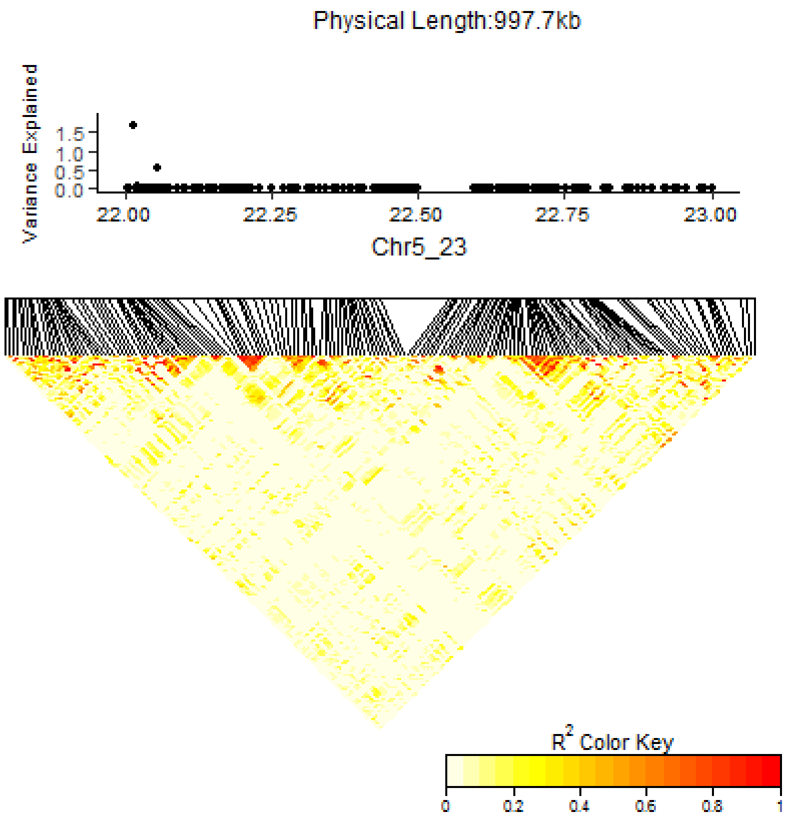

Figure 4q. Manhattan plot of the variance explained by each SNP in the window Chr5_23 for $dEBV_v$ and linkage disequilibrium (LD; r^2)

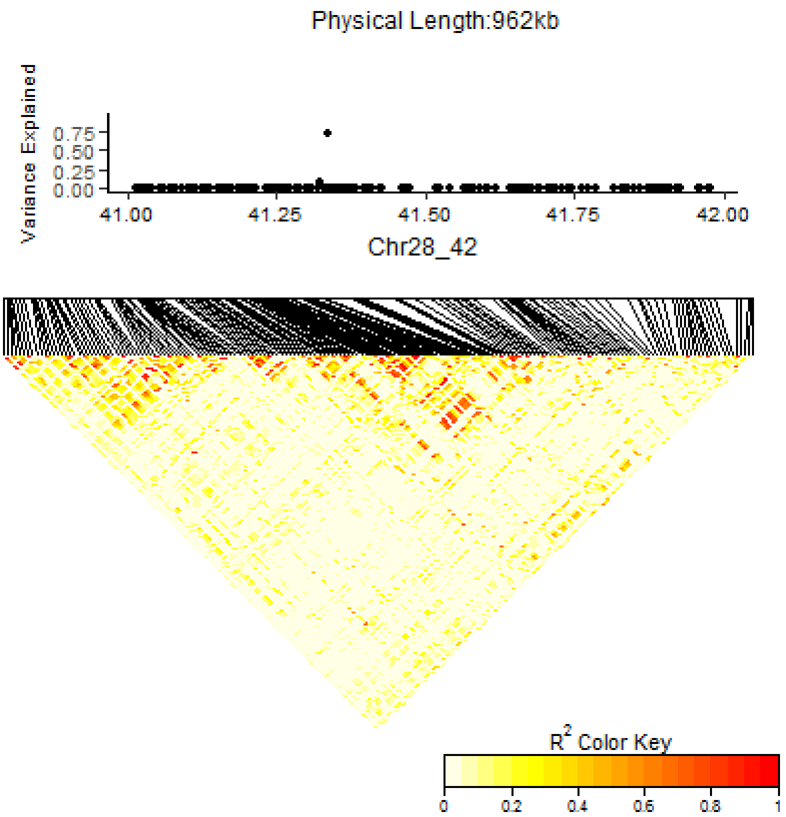

Figure 4r. Manhattan plot of the variance explained by each SNP in the window Chr28_42 for $dEBV_v$ and linkage disequilibrium (LD; r^2)

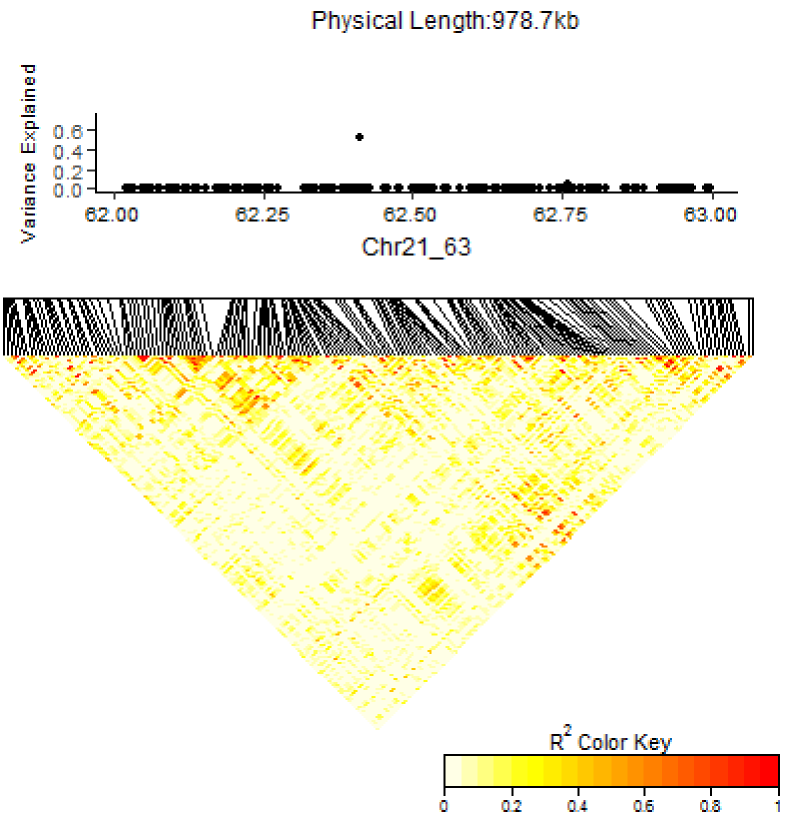

Figure 4s. Manhattan plot of the variance explained by each SNP in the window Chr21_63 for $dEBV_v$ and linkage disequilibrium (LD; r^2)

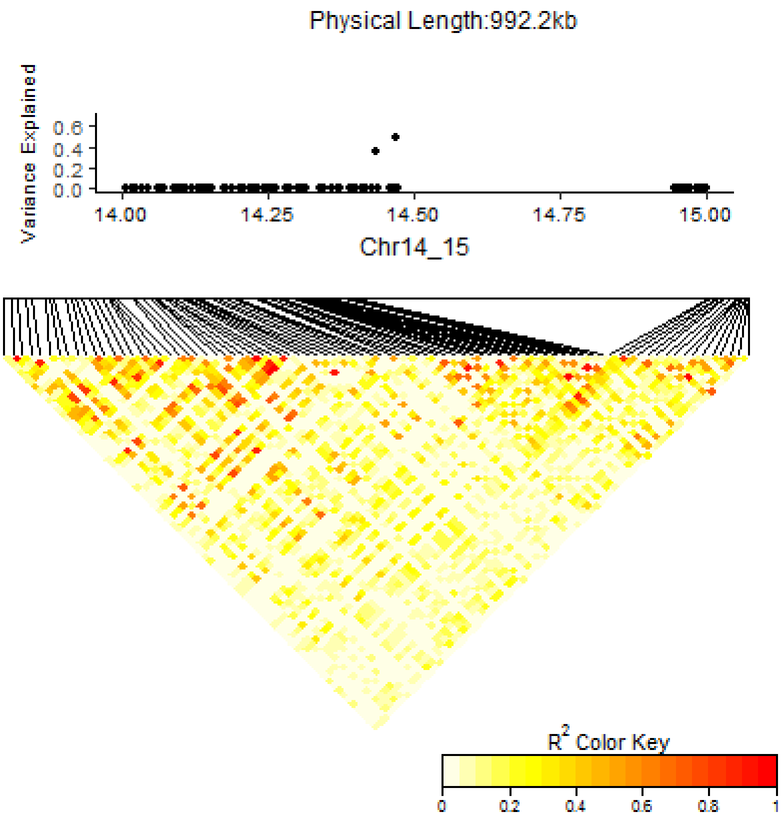

Figure 4t. Manhattan plot of the variance explained by each SNP in the window Chr14_15 for $dEBV_v$ and linkage disequilibrium (LD; r^2)
